# Research trends of tumor-associated macrophages in colorectal cancer: a bibliometric analysis

**DOI:** 10.1097/JS9.0000000000004422

**Published:** 2026-02-23

**Authors:** Yuxin Long, Xiangxu Wang, Xiaoting Chao, Yue Yang, Shuo Jin, Hong-Mei Zhang

**Affiliations:** a The Second Clinical Medical College, Shanxi University of Chinese Medicine, Xianyang, China; b Department of Clinical Oncology, Xijing Hospital, Air Force Military Medical University, Xi’an, China

**Keywords:** bibliometric, CRC, future trend, research topic, TAMs

## Abstract

**Background:**

Colorectal cancer (CRC) represents the third most prevalent malignancy worldwide and accounts for the second-highest cancer-related mortality rate. Accumulating evidence over the past decade has established the pivotal role of tumor-associated macrophages (TAMs) in CRC tumorigenesis and disease progression. This study employs bibliometric analysis to delineate the research landscape and emerging frontiers in CRC TAMs investigation.

**Methods:**

We systematically retrieved publications indexed in the Web of Science Core Collection from database inception through 14 April 2025, applying predefined inclusion and exclusion criteria. Using VOSviewer and CiteSpace, we conducted comprehensive visual analyses of the CRC TAMs research domain, including country contributions, institutional productivity, annual publication trends, and journal distributions.

**Results:**

Our bibliometric analysis identified 2861 publications on CRC TAMs worldwide, with a consistent upward publication trend since 2018. Geospatial analysis revealed that China and the United States are the predominant contributing nations, with Sun Yat-sen University being the most productive institution. Keyword co-occurrence mapping identified five predominant research clusters: CRC biology, TAMs characterization, patient survival outcomes, disease progression mechanisms, and metastatic processes. Four key research directions emerged: (1) single-cell RNA sequencing for TAMs heterogeneity analysis, (2) PD-1/PD-L1 checkpoint interactions with TAMs, (3) molecular mechanisms underlying macrophage polarization, and (4) chemotherapeutic modulation of TAMs functionality (particularly irinotecan). These advances provide mechanistic insights into CRC TAMs biology and may facilitate (1) the discovery of novel diagnostic biomarkers, (2) precision therapeutic strategies, and (3) personalized prognostic frameworks.

**Conclusion:**

Bibliometric analysis has highlighted the global landscape of research on CRC TAMs. Future research directions in CRC TAMs are likely to center on the integration of multi-omics, in-depth investigation of the tumor microenvironment, targeting TAMs, application of machine learning and artificial intelligence, and the development of personalized treatment strategies for CRC.

## Introduction

Colorectal cancer (CRC) represents a leading global health burden^[1]^, ranked as the third most prevalent malignancy worldwide, with its progression intrinsically linked to the tumor microenvironment (TME)^[2,3]^. As pivotal immunomodulators in the CRC TME, tumor-associated macrophages (TAMs) orchestrate tumor progression via proliferation, invasion, angiogenesis, immune evasion, and metabolic reconfiguration.

Macrophages exhibit polarization plasticity, differentiating into M1 (pro-inflammatory, tumoricidal) and M2 (anti-inflammatory, tumor-promoting) phenotypes^[4,5]^. Current TAM-directed therapeutic strategies include: (1) inhibition of CCR2-mediated precursor recruitment^[6]^, (2) selective depletion of M2-polarized subsets^[7]^, (3) enhancement of phagocytic capacity^[8]^, (4) phenotypic reprogramming (M2-to-M1)^[9]^, and (5) blockade of monocyte trafficking^[10]^.

Despite significant advances, a comprehensive scientometric evaluation of the field’s evolution remains lacking. Bibliometric analysis enables quantitative assessment of research dynamics, academic influence, and collaboration patterns. We employ CiteSpace (v6.2.R4) and VOSviewer (v1.6.18) to: (1) map global collaboration networks, (2) analyze temporal publication trends, (3) identify emerging research frontiers, and (4) highlight translational challenges.

## Materials and methods

### Search strategy

We conducted a systematic search in the Web of Science Core Collection (WoSCC), the most authoritative global citation database, to identify relevant publications. Our search strategy employed the following Boolean query: (TS = (“Colorectal Neoplasms” OR “Colorectal Neoplasm” OR “Neoplasm, Colorectal” OR “Colorectal Tumors” OR “Colorectal Tumor” OR “Tumor, Colorectal” OR “Tumors, Colorectal” OR “Neoplasms, Colorectal” OR “Colorectal Cancer” OR “Cancer, Colorectal” OR “Cancers, Colorectal” OR “Colorectal Cancers” OR “Colorectal Carcinoma” OR “Carcinoma, Colorectal” OR “Carcinomas, Colorectal” OR “Colorectal Carcinomas”) AND (TS = (macrophage * OR “Tumor-Associated Macrophages” OR “TAMs” OR “M1” OR “M2”)). The search encompassed all available records from database inception through 14 April 2025.

### Data selection

**Figure 1.**
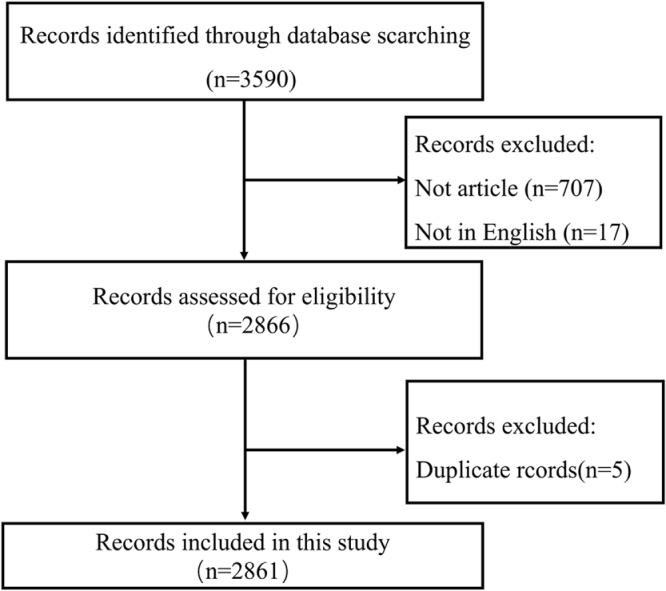
Flowchart of CRC TAMs literature screening.

The selection criteria and literature screening process of this study are depicted in Figure 1. Initially, we input the search terms to conduct the primary search. Subsequently, two researchers independently reviewed the publications identified in the initial search. For inclusion criteria, the document type was restricted to English-language Articles published from the inception of WOS to 14 April 2025, and the publications had to be sourced from the Science Citation Index Expanded (WoSCC Citation Index Expanded, SCI-E) database. Exclusion criteria included: non-English Articles; non-research-type documents, such as letters, editorials, reviews, and conference abstracts; publications with missing data fields (e.g., no authors, publication year, or keywords); and duplicate publications. After multiple rounds of screening, a total of 2861 relevant studies were ultimately included.

### Data analysis

We performed network analyses of institutional, national, and journal collaborations using VOSviewer (v1.6.20) and Scimago Graphica (v1.0.49). Network visualizations represent entities as nodes (countries/institutions/authors), where node size correlates with publication output and connection thickness reflects collaboration intensity. For knowledge domain analysis, we utilized CiteSpace (v6.4.1) for keyword co-occurrence and co-citation network analyses. By transforming the latent knowledge information within CRC TAMs literature into a panoramic view, this study aims to provide researchers in the field of CRC TAMs with a clear and comprehensive perspective. This will facilitate a better understanding of the current research landscape, enable the prediction of future developmental trajectories, and foster scientific collaboration on a global scale^[1]^.

HIGHLIGHTS

- Large-scale bibliometric analysis: This study analyzed 2861 publications on tumor-associated macrophages (TAMs) in colorectal cancer (CRC), revealing global research trends and emerging frontiers from database inception to 2025, with a total citation count reflecting significant academic impact.
- Translational and clinical implications: The findings underscore TAMs as pivotal therapeutic targets, offering potential for novel diagnostic biomarkers, precision therapies, and personalized prognostic frameworks in CRC management.
- Future research directions: The study highlights emerging trends such as multi-omics integration, in-depth tumor microenvironment exploration, AI-driven predictive modeling, and tailored therapeutic strategies, providing a roadmap for advancing CRC TAMs research and clinical applications.

## Results

### Publication trends and geographic distribution

**Figure 2.**
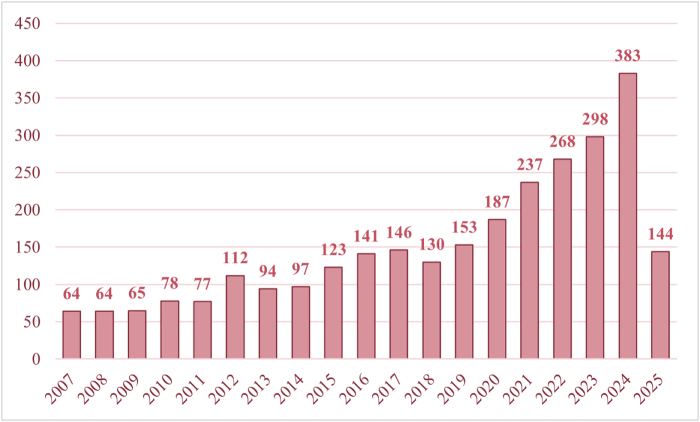
Year of publication of CRC TAMs literature.

**Figure 3.**
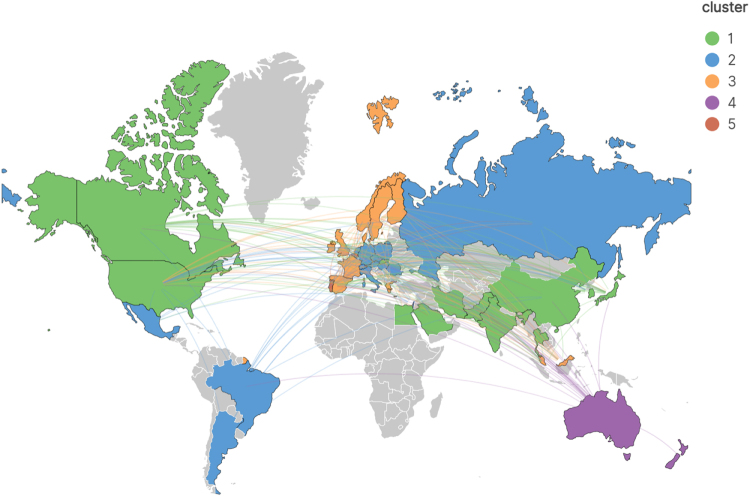
Diagram of countries/regions map.

**Figure 4.**
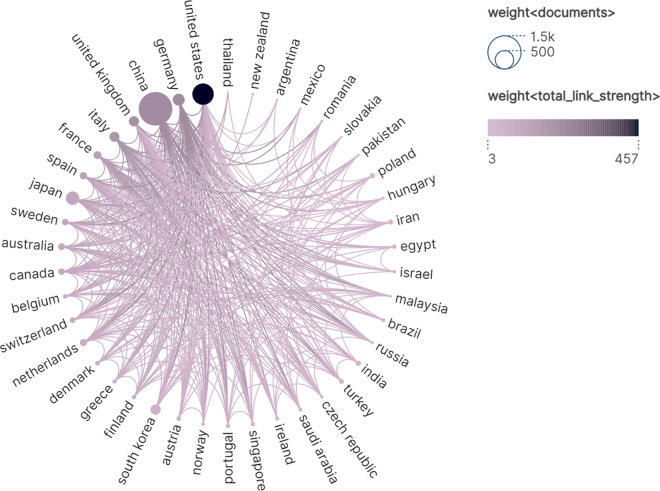
Strength of international collaboration.

Our systematic search identified 3590 records published between database inception and 14 April 2025. After excluding 724 non-research publications (reviews, conference abstracts, letters, and works in progress), 2866 articles remained for analysis. Following deduplication using CiteSpace, 2861 studies met our inclusion criteria for bibliometric analysis (Fig. 1). Subsequently, we summarized the annual publication output characteristics of CRC TAMs. The overall trend in annual publication volume has been increasing, particularly from 2018 to 2024, with a continuous rise in cumulative publications. The peak was reached in 2024, with 383 publications accounting for 13.39% of the total (Fig. 2), indicating that the field of CRC TAMs is gradually gaining more attention. In the first quarter of 2025, 144 publications were released, suggesting that the number of publications in 2025 is likely to surpass that of 2024. We analyzed the contributions of different countries to CRC TAMs research and used VOSviewer and Scimago Graphica software to investigate the publishing countries/regions. To avoid ambiguity in country names, we followed the naming conventions of Scimago Graphica, consolidating “England,” “Scotland,” “Wales,” and “Northern Ireland” into “the United Kingdom.” With a threshold of 5, we identified that researchers from 42 countries/regions have participated in CRC TAMs research, forming 5 major clusters (Fig. 3). China published the most articles (*N* = 1381), followed by the United States (*N* = 557) and Japan (*N* = 220) (Table 1). Despite China’s highest publication output, the United States has the most frequent collaborations with other countries, indicating that China still has room for improvement in international communication and cooperation (Fig. 4). Therefore, future efforts should focus on strengthening international collaboration to promote information sharing and academic exchange, thereby propelling the in-depth development of TAMs and CRC research.

**Table 1:** Top 10 contributing countries (ranked by publication count).

Number	Country	Articles	Freq = articles/total articles
1	China	1381	0.483
2	United States	557	0.195
3	Japan	220	0.077
4	Germany	173	0.060
5	South Korea	131	0.046
6	United Kingdom	125	0.044
7	Italy	122	0.043
8	France	75	0.026
9	Spain	62	0.022
10	Australia	58	0.020

### Institutional and author contributions

To visually illustrate collaborations among institutions and research directions, we used VOSviewer software to analyze publishing institutions. The analysis revealed that 3642 institutions have been involved in CRC TAMs research. Sun Yat-sen University, Zhejiang University, and Fudan University emerged as key institutions in this field. The top 10 institutions are summarized in Table 2. Among them, Sun Yat-sen University leads with 130 published papers, followed by Zhejiang University with 89 papers and Fudan University with 81 papers.

**Table 2:** Summary of the top 10 institutions in the field of CRC TAMs.

Number	Institution	Publications	Total citations	Connectivity strength
1	Sun Yat-sen University	103	2733	190
2	Zhejiang University	89	2959	189
3	Fudan University	81	3763	161
4	Shanghai Jiao Tong University	76	2789	149
5	Nanjing Medical University	73	2459	162
6	Southern Medical University	53	1676	106
7	Chinese Academy of Sciences	48	2421	175
8	Nanjing University Chinese Medical	45	534	98
9	China Medical University	43	815	84
10	Peking University	40	2268	78

### Keywords co-occurrence, clusters, and bursts

**Figure 5.**
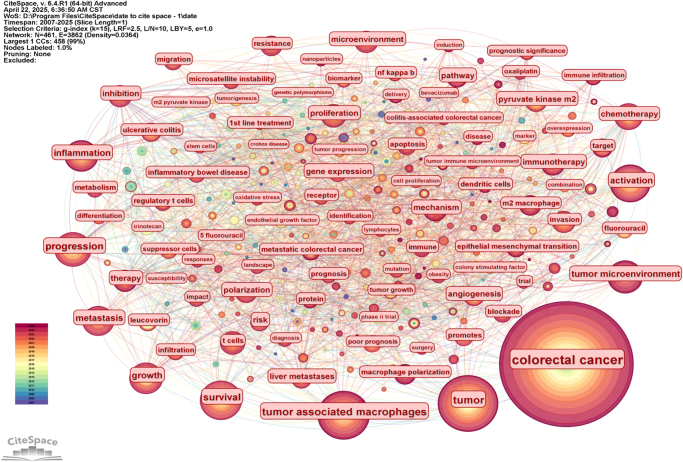
Network visualization of co-occurring keywords in CRC TAM domains.

**Figure 6.**
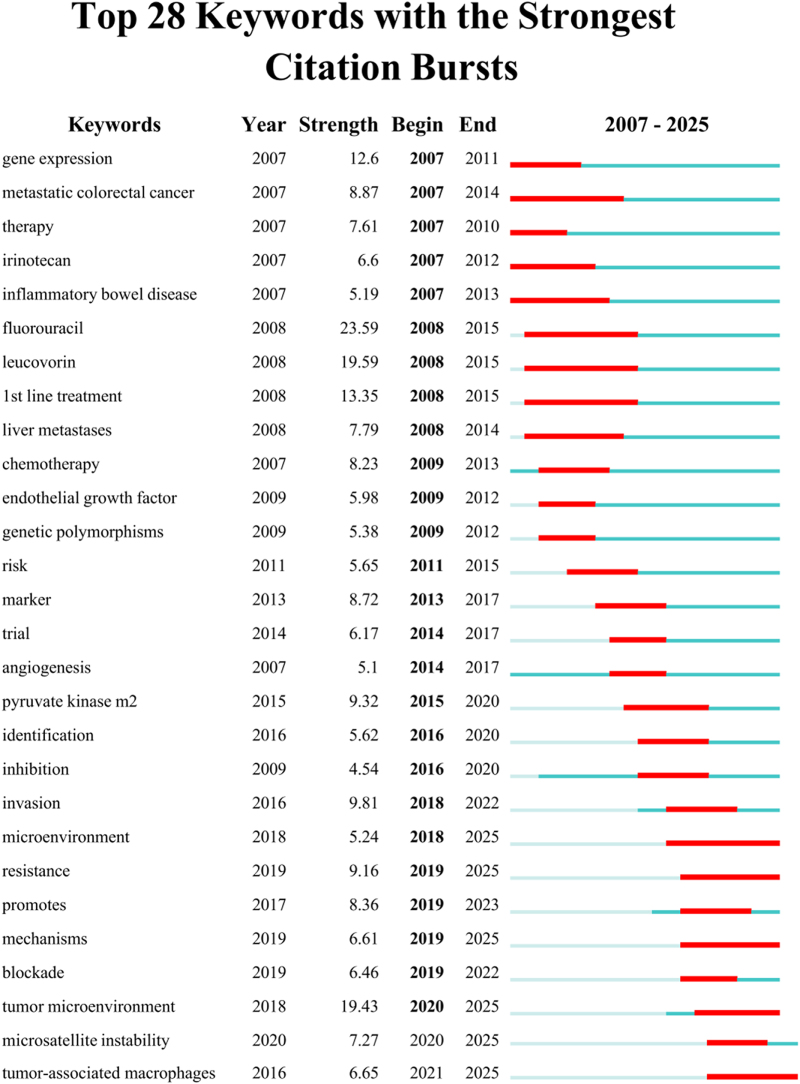
Most explosive keywords for CRC TAMs citations.

Using CiteSpace software, we analyzed 461 keywords in the literature (Figs 5 and 6). The most frequent keywords were CRC (*N* = 1967), tumor (*N* = 500), TAMs (*N* = 406), and survival (*N* = 276). Keywords related to tumor characteristics and progression included progression (*N* = 258), activation (*N* = 239), metastasis (*N* = 206), growth (*N* = 188), TME (*N* = 180), and proliferation (*N* = 102).

**Figure 7.**
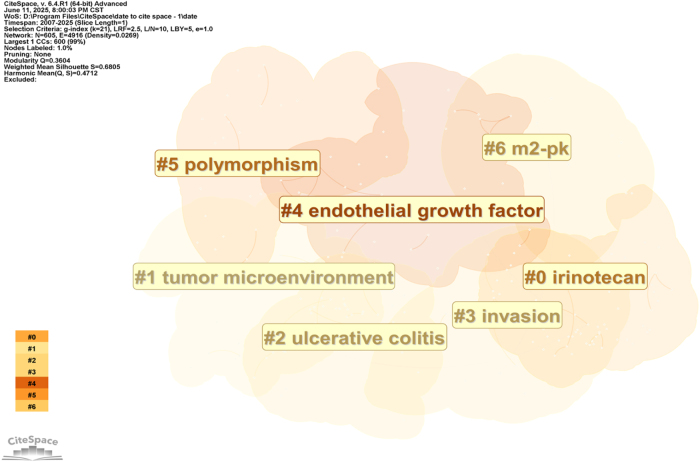
Network visualization of CRC TAMs keyword clustering.

Keywords related to biomarker identification and expression included gene expression (*N* = 107), differentiation (*N* = 110), and angiogenesis (*N* = 102). To identify research hotspots, we used the log-likelihood ratio method to cluster keywords in CRC TAMs articles, resulting in seven distinct clusters (Fig. 7, Table 3). Cluster 0 focused on irinotecan and chemotherapeutic agents (e.g., capecitabine, oxaliplatin, leucovorin) in metastatic CRC, exploring their impact on the TME and potential regulatory mechanisms on TAMs. Cluster 1 examined the TME, involving TAMs and tumor immunotherapy, to uncover the role of CRC TAMs and their regulatory mechanisms in immunotherapy. Cluster 2 investigated ulcerative colitis (including inflammatory bowel disease, inflammation, colitis) to explore macrophage function and regulatory mechanisms in chronic inflammation. Cluster 3 focused on invasion (foxml, pkm2, migration) in CRC, studying their mechanisms and TAMs roles in tumor invasion and metastasis to provide new treatment ideas. Cluster 4 examined vascular endothelial growth factor’s impact on CRC (tumor proliferation, VEGF, tumor markers, angiogenesis) and CRC TAMs role in tumor angiogenesis. Cluster 5 focused on polymorphism (obesity, GSTM1, risk, body mass index) and macrophage functional differences and their impact on tumor occurrence and development. Cluster 6 centered on m2-pk (long non-coding RNA, adenoma, carcinoembryonic antigen, screening) and investigated CRC TAMs role in tumor metabolism, early screening, and diagnosis.

**Table 3:** Keyword cluster analysis of CRC TAMs papers.

Cluster	Size	Silhouette	Mean year	Label (Log-likelihood ratio [LLR])
0	117	0.752	2013	irinotecan (125.86, 1.0E-4); capecitabine (117.69, 1.0E-4); oxaliplatin (96.51, 1.0E-4); metastatic colorectal cancer (92.89, 1.0E-4); leucovorin (72.86, 1.0E-4)
1	114	0.548	2017	tumor microenvironment (66.26, 1.0E-4); immunotherapy (52.89, 1.0E-4); tumor-associated macrophages (37.45, 1.0E-4); tumor associated macrophages (34.12, 1.0E-4); cancer immunotherapy (27.95, 1.0E-4)
2	108	0.668	2014	ulcerative colitis (88.15, 1.0E-4); inflammatory bowel disease (71.75, 1.0E-4); inflammation (69.66, 1.0E-4); colitis (48.8, 1.0E-4); macrophage (46.42, 1.0E-4)
3	104	0.644	2016	invasion (60.97, 1.0E-4); foxm1 (46.75, 1.0E-4); pkm2 (37.08, 1.0E-4); migration (35.99, 1.0E-4); colorectal cancer (34.66, 1.0E-4)
4	68	0.735	2010	endothelial growth factor (26.88, 1.0E-4); tumor growth (24.18, 1.0E-4); vegf (22.25, 1.0E-4); tumor markers (20.6, 1.0E-4); angiogenesis (17.92, 1.0E-4)
5	61	0.736	2014	polymorphism (72.63, 1.0E-4); obesity (65.31, 1.0E-4); gstm1 (62.91, 1.0E-4); risk (62.65, 1.0E-4); body mass index (60.61, 1.0E-4)
6	28	0.852	2015	m2-pk (22.3, 1.0E-4); long non-coding rna (20.62, 1.0E-4); adenomas (16.19, 1.0E-4); carcinoembryonic antigen (14.02, 0.001); screening (13.74, 0.001)

**Figure 8.**
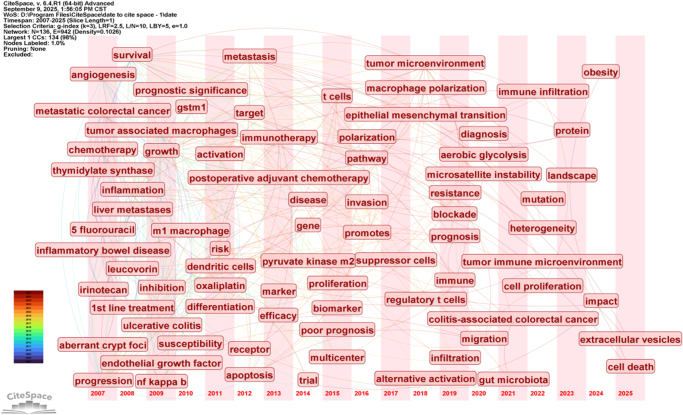
Timeline mapping of CRC TAMs domain keywords.

**Figure 9.**
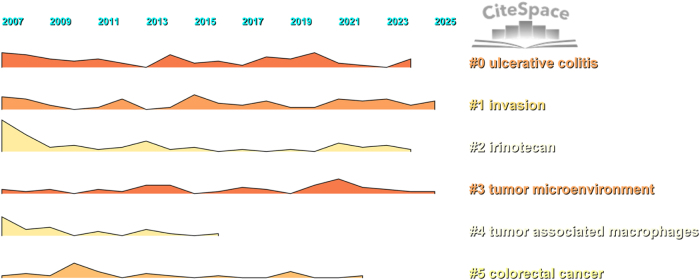
Timeline mapping for CRC TAMs keyword clustering.

**Figure 10.**
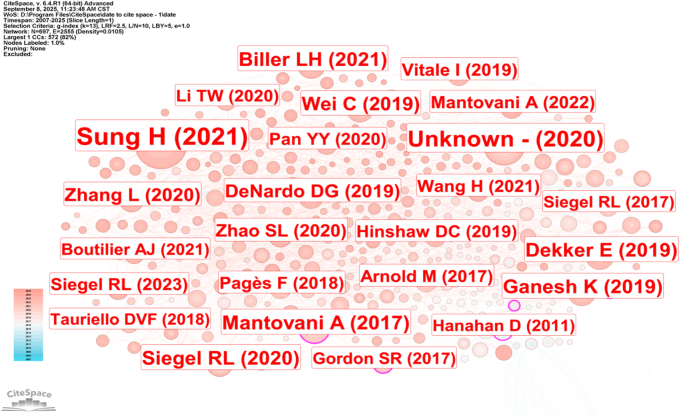
Co-citation visualization analysis of CRC TAMs literature.

The keyword timeline map shows the evolution of high-frequency keywords. Through CiteSpace burst analysis of keywords (Fig. 8), with a 1-year time span, research foci and hotspots varied across different periods. Among the top 28 burst keywords, those from 2007 to 2009 included gene expression, metastatic CRC, and therapy; from 2010 to 2015, risk and biomarkers emerged; and from 2016 to 2020, TAMs, identification, and invasion were highlighted. Additionally, keyword timeline analysis (Fig. 9) and cluster timeline analysis (Fig. 10) were performed. The analysis indicated that from 2007 to 2013, the primary focus was on macrophages’ impact on chemotherapy, particularly the application of oxaliplatin, irinotecan, and fluorouracil. In 2018, CRC TAMs were proposed as a therapeutic target. From 2019 to 2025, the focus shifted to the tumor immune microenvironment and microsatellite-unstable CRC. In mechanism research, early attention focused on endothelial growth factor, NF-κB, etc. Since 2021, the focus has been on the gut microbiota, and research has expanded beyond macrophage polarization to a more comprehensive examination of TME. Cluster timeline analysis of keywords shows that ulcerative colitis and invasion have consistently been hotspots in CRC TAMs research. Irinotecan received the most attention in 2007, which then gradually decreased. TME was the most focused on during 2020–2021 (Fig. 10).

### Co-cited articles and co-cited reference cluster analysis

**Figure 11.**
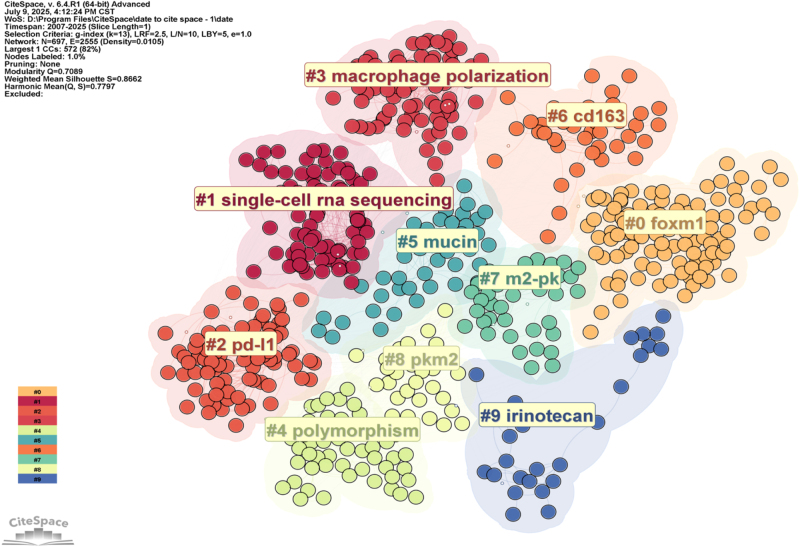
Visualization of CRC TAMs co-citation literature clustering.

Co-citation analysis is a method that reveals the interconnections among literature by detecting other articles that cite the same document^[11]^. We conducted a co-citation analysis of 697 CRC TAMs articles using CiteSpace (Fig. 11) and identified the 10 most highly co-cited articles (Table 4). The largest node corresponds to an article published in 2021 in *CA: A Cancer Journal for Clinicians*, titled “Global cancer statistics 2020: GLOBOCAN estimates of incidence and mortality worldwide for 36 cancers in 185 countries,” with the highest citation count (229). An article published in 2019 in *Nature Reviews Gastroenterology & Hepatology* emphasized immunotherapy for mismatch-repair-deficient and microsatellite instability-high CRC (dMMR-MSI-H) versus mismatch-repair-proficient and low microsatellite instability CRC (pMMR-MSI-L)^[12]^. Research shows that PD-1 inhibitors demonstrate significant efficacy in dMMR-MSI-H CRC (objective response rate of 35–55%), yet approximately 40–50% of patients still exhibit primary resistance^[13,14]^. Articles published in 2017 in *Nature Reviews Clinical Oncology*^[15]^ and in 2019 in *Nature Reviews Immunology*^[16]^ highlighted the role of TAMs in various tumor treatments and emerging macrophage-targeting therapies. Current therapeutic strategies targeting TAMs face the following key issues: (1) Insufficient targeting specificity, as existing methods for eliminating TAMs often nonspecifically affect the mononuclear-macrophage system, potentially disrupting tissue homeostasis. Developing technologies that selectively target TAMs while preserving resident macrophages is crucial for ensuring therapeutic safety^[17,18]^; (2) Limitations of therapeutic strategies, including cytokine/receptor modulation (prone to immune-related adverse reactions), inhibition of TAMs recruitment (which may accelerate metastasis), enhancement of phagocytic function (e.g., targeting the CD47-SIRPα pathway), and promotion of M1-type polarization (with complex mechanisms and significant individual variability); (3) Insufficient scientific understanding, as the simplified M1/M2 dichotomy model is controversial, the pro-tumor role of M2-type TAMs is not absolute, phenotypic plasticity is regulated by multiple factors, and there are tumor type and individual differences^[19]^. Future breakthroughs should focus on using multi-omics technologies to elucidate TAMs heterogeneity, developing new methods for precise regulation of the microenvironment, establishing individualized treatment prediction systems, and exploring combination therapy strategies. The clinical translation of TAM-targeting therapies will require multidisciplinary collaboration integrating basic research, bioengineering, and clinical medicine to systematically address the complex regulatory networks of macrophages in the TME. An article published in 2020 in *Cell*^[20]^ indicated that anti-CSF1R treatment can selectively eliminate pro-inflammatory macrophages while preserving pro-angiogenic/pro-tumorigenic macrophage subpopulations, suggesting that targeting TAMs should consider subpopulation specificity and the development of more precise combination strategies. Traditionally, macrophages have been divided into M1 and M2 types, but this classification does not fully reflect the diversity of macrophages in the TME^[21,22]^, and there is an overlap in the gene expression profiles of the two types^[23]^. In recent years, single-cell RNA sequencing has revealed the high heterogeneity of TAMs, identifying multiple functionally distinct subpopulations (such as C1Q^+^ TAMs). Studies have shown that targeting the upstream and downstream mechanisms mediated by C1Q^+^ TAMs can alleviate immune suppression and enhance the efficacy of immune checkpoint blockade therapy^[23,24,25,25,26]^. These findings indicate that specific TAM subpopulations can serve as potential therapeutic targets, offering new directions for cancer treatment. Future research should further elucidate the functional diversity of TAMs and establish a TAM classification system for tumors such as CRC based on molecular characteristics to advance precision therapies targeting specific subpopulations.

**Table 4:** The top 10 co-cited articles related to CRC TAMs.

Year	Citations	First author	Journal	Title
2021	229	Sung H	*CA Cancer J Clin*	Global cancer statistics 2020: GLOBOCAN estimates of incidence and mortality worldwide for 36 cancers in 185 countries^[27]^
2020	141	Bray F	*CA Cancer J Clin*	Global cancer statistics 2018: GLOBOCAN estimates of incidence and mortality worldwide for 36 cancers in 185 countries^[28]^
2017	71	Mantovani A	*Nat Rev Clin Oncol*	Tumour-associated macrophages as treatment targets in oncology^[15]^
2019	70	Dekker E	*Lancet*	Colorectal cancer^[29]^
2019	68	Ganesh K.	*Nat Rev Gastroenterol Hepatol*	Immunotherapy in colorectal cancer: rationale, challenges and potential^[12]^
2020	66	Siegel RL	*CA Cancer J Clin*	Cancer statistics, 2020^[30]^
2021	65	Biller LH	*JAMA*	Diagnosis and treatment of metastatic colorectal cancer: a review^[31]^
2019	62	Wei C	*Mol Cancer*	Crosstalk between cancer cells and tumor associated macrophages is required for mesenchymal circulating tumor cell-mediated colorectal cancer metastasis^[32]^
2019	53	DeNardo DG	*Nat Rev Immunol*	Macrophages as regulators of tumour immunity and immunotherapy^[16]^
2020	53	Zhang L	*Cell*	Single-cell analyses inform mechanisms of myeloid-targeted therapies in colon cancer^[20]^

We conducted clustering analysis on the literature (Fig. 11, Table 5) and identified 10 major research areas in CRC TAMs. Cluster 0: Focuses on FOXM1, exploring the specific functions and regulatory mechanisms of CRC TAMs in the TME. Cluster 1: Concentrates on single-cell sequencing studies of CRC TAMs, revealing interactions among CRC TAMs and their spatial heterogeneity. Cluster 2: Investigates PD-L1, exploring the relationship between CRC TAMs and immune checkpoint inhibitors and their role in immunotherapy. Cluster 3: Studies macrophage polarization and its regulatory roles in the TME. Cluster 4: Examines the impact of genetic polymorphisms on CRC TAMs. Cluster 5: Investigates functional differences of CRC TAMs under mucin polymorphisms and their impact on the tumor immune microenvironment and patient prognosis. Cluster 6: Studies the function of CD163^+^ TAMs in CRC and their relationship with lymph node metastasis. Cluster 7: Focuses on the application of pyruvate kinase 2 in CRC screening, exploring its metabolic regulatory role in the TME. Cluster 8: Investigates the metabolic regulation of pyruvate kinase 2 and pyruvate kinase 1, focusing on their functional differences and regulatory mechanisms in TAMs. Cluster 9: Highlights the application of irinotecan in CRC treatment and its impact on the TME (including TAMs).

**Table 5:** Reference clustering of CRC TAMs papers.

Cluster	Size	Silhouette	Mean year	Label (LLR)
0	103	0.823	2014	foxm1 (31.96, 1.0E-4); gene therapy (7.96, 0.005); e-cadherin (7.96, 0.005); 5-fluorouracil (4.51, 0.05); hypoxia (4.44, 0.05)
1	81	0.868	2021	single-cell rna sequencing (26.53, 1.0E-4); scrna-seq (17.07, 1.0E-4); network pharmacology (9.81, 0.005); spatial transcriptomics (9.81, 0.005); colorectal cancer (6.63, 0.05)
2	81	0.818	2018	pd-l1 (15.68, 1.0E-4); immune checkpoint inhibitors (14.02, 0.001); fusobacterium nucleatum (12.81, 0.001); immunotherapy (11.15, 0.001); tumor-associated macrophages (8.97, 0.005)
3	80	0.747	2020	macrophage polarization (17.78, 1.0E-4); extracellular vesicles (10.79, 0.005); cxcl12 (9.7, 0.005); tumor microenvironment (9, 0.005); exosome (6.46, 0.05)
4	54	0.903	2011	polymorphism (26.64, 1.0E-4); gstm1 (21.3, 1.0E-4); carcinogenesis (15.96, 1.0E-4); meta-analysis (15.96, 1.0E-4); caco-2 (10.63, 0.005)
5	43	0.96	2006	mucin (14.25, 0.001); cd14 (14.25, 0.001); survival (10.3, 0.005); macrophages (9.18, 0.005); immune response (8.82, 0.005)
6	39	0.895	2017	cd163 (14.59, 0.001); lymph node metastasis (11.05, 0.001); TAMss (10.01, 0.005); immunotherapy (8.51, 0.005); cancer stem cells (7.87, 0.01)
7	37	0.995	2006	m2-pk (24.88, 1.0E-4); colorectal cancer screening (16.54, 1.0E-4); faecal (16.54, 1.0E-4); polyps (16.54, 1.0E-4); pyruvate kinase (12.76, 0.001)
8	28	0.98	2012	pkm2 (31.43, 1.0E-4); pyruvate kinase m2 (19.96, 1.0E-4); proliferation (15.36, 1.0E-4); pkm1 (14.63, 0.001); alternative splicing (14.63, 0.001)
9	26	0.97	2004	irinotecan (26.45, 1.0E-4); rectal cancer (12.08, 0.001); gemcitabine (8.76, 0.005); synchronous disease (8.76, 0.005); pharmacokinetics (8.76, 0.005)

## Discussion

This study employs bibliometric tools, including CiteSpace, VOSviewer, and Scimago Graphica, to analyze literature related to CRC TAMs, aiming to elucidate research trends and key findings in this domain. Through extensive retrieval and analysis of a vast array of relevant literature, several crucial conclusions have been reached.

First, a significant increase in CRC TAMs research has been observed in recent years. As the demand for personalized treatment of CRC TAMs continues to increase, the academic community’s focus on this area has intensified. This indicates that the significance of TAMs in CRC research is gradually being acknowledged, with relevant studies being widely conducted globally. The national analysis reveals that China, the United States, and Japan currently dominate this field, which is closely associated with their advantages in research funding, research institutions, technological level, clinical resources, and international cooperation. The institutional analysis shows that high-productive research institutions, with Sun Yat-sen University, Zhejiang University, and Fudan University as core contributors, have established relatively stable collaborative groups.

Keyword analysis has revealed the research interests and directions of investigators in searching for new biomarkers, evaluating therapeutic strategies, and predicting patient prognosis. Moreover, keyword burst and timeline analysis indicate that since 2007, certain keywords such as gene, invasion, risk, biomarker, and treatment have consistently garnered the attention of scholars. In recent years, researchers have shown increasing interest in keywords such as colitis-associated CRC, gut microbiota, therapeutic targets, and TME, and have demonstrated significant interest in predicting CRC prognosis and treatment outcomes.

Literature clustering analysis reveals that CRC TAMs research primarily focuses on several key areas, including irinotecan, TME, ulcerative colitis, and vascular endothelial growth factor. These studies aim to improve treatment outcomes for CRC patients, facilitate personalized treatment, and enhance prognostic assessment. In this study, we analyzed institutions, countries, keywords, and co-cited literature in the field of CRC TAMs. Our results show that current research is particularly concentrated on immune checkpoint biomarkers (e.g., PD-L1 expression and immune cell infiltration levels), factors influencing CRC development (e.g., macrophage polarization, foxm1, mucin, cd163, m2-pk), and genomic biomarkers (e.g., mutations, gene expression, and genomic structural variations). These research foci help to gain a deeper understanding of the biological processes of CRC TAMs, thereby providing more precise biomarkers for diagnosis, treatment, and prognosis. By analyzing institutions, countries, highly cited, and recently highly used literature of CRC TAMs articles, we predict that future CRC TAMs research will shift towards multi-omics integration (genomics, transcriptomics, proteomics, metabolomics)^[33,34]^, detailed exploration of the TME (including different cell types, cytokines, and cell interactions), targeting TAMs (related to CRC drug sensitivity and resistance)^[35]^, using machine learning and artificial intelligence (data mining, predictive modeling), and developing personalized treatment strategies.

We encourage young researchers to engage in interdisciplinary learning and collaboration, master new technologies and methods, enhance data analysis skills, stay updated with the latest research trends, and effectively address the challenges and opportunities in the field of CRC TAMs.

## Limitations

### Data sources

Our study primarily relies on published papers within the WoSCC database. This approach may result in the exclusion of unpublished studies or grey literature, thereby potentially compromising the comprehensiveness and accuracy of our findings.

### Data analysis

Although we employed bibliometric methods for analysis, these methods inherently possess certain limitations. We were unable to access raw data from the literature and had to depend solely on the information provided in the papers. Moreover, our analysis was constrained by the selected keywords and the defined time frame. Data interpretation: Our interpretation of publication counts, citation frequencies, and keywords may be somewhat subjective. Consequently, different researchers might arrive at divergent conclusions.

## Data Availability

Original contributions are included in the article. Further inquiries may be directed to the corresponding authors.

## References

[1] Riaz AA Ginimol M Rasha R. Transparency in the reporting of Artificial Intelligence – the TITAN Guideline [J]. Prem J Sci 2025;10:100082.

[2] Elhanani O Ben-Uri R Keren L. Spatial profiling technologies illuminate the tumor microenvironment [J]. Cancer Cell 2023;41:404–20. 36800999 10.1016/j.ccell.2023.01.010

[3] Cassetta L Pollard JW. Targeting macrophages: therapeutic approaches in cancer [J]. Nat Rev Drug Discov 2018;17:887–904. 30361552 10.1038/nrd.2018.169

[4] Wang H Wang X Zhang X. The promising role of tumor-associated macrophages in the treatment of cancer [J]. Drug Resist Updat 2024;73:101041. 38198845 10.1016/j.drup.2023.101041

[5] Anderson NR Minutolo NG Gill S. Macrophage-based approaches for cancer immunotherapy [J]. Cancer Res 2021;81:1201–08. 33203697 10.1158/0008-5472.CAN-20-2990

[6] Myers KV Amend SR Pienta KJ. Targeting Tyro3, Axl and MerTK (TAM receptors): implications for macrophages in the tumor microenvironment [J]. Mol Cancer 2019;18:94. 31088471 10.1186/s12943-019-1022-2 PMC6515593

[7] Lee C Jeong H Bae Y. Targeting of M2-like tumor-associated macrophages with a melittin-based pro-apoptotic peptide [J]. J Immunother Cancer 2019;7:147. 31174610 10.1186/s40425-019-0610-4 PMC6555931

[8] Chen H Yang Y Deng Y. Delivery of CD47 blocker SIRPα-Fc by CAR-T cells enhances antitumor efficacy [J]. J Immunother Cancer 2022;10:e003737. 10.1136/jitc-2021-003737 PMC8811602 35110357

[9] Figueiredo P Lepland A Scodeller P. Peptide-guided resiquimod-loaded lignin nanoparticles convert tumor-associated macrophages from M2 to M1 phenotype for enhanced chemotherapy [J]. Acta Biomater 2021;133:231–43. 33011297 10.1016/j.actbio.2020.09.038

[10] Hourani T Holden JA Li W. Tumor associated macrophages: origin, recruitment, phenotypic diversity, and targeting [J]. Front Oncol 2021;11:788365. 34988021 10.3389/fonc.2021.788365 PMC8722774

[11] Wang X Dong T Li X. Global biomarker trends in Parkinson’s disease research: a bibliometric analysis [J]. Heliyon 2024;10:e27437. 38501016 10.1016/j.heliyon.2024.e27437 PMC10945172

[12] Ganesh K Stadler ZK Cercek A. Immunotherapy in colorectal cancer: rationale, challenges and potential [J]. Nat Rev Gastroenterol Hepatol 2019;16:361–75. 30886395 10.1038/s41575-019-0126-x PMC7295073

[13] Cercek A Lumish M Sinopoli J. PD-1 blockade in mismatch repair-deficient, locally advanced rectal cancer [J]. N Engl J Med 2022;386:2363–76. 35660797 10.1056/NEJMoa2201445 PMC9492301

[14] André T Lonardi S Wong KYM. Nivolumab plus low-dose ipilimumab in previously treated patients with microsatellite instability-high/mismatch repair-deficient metastatic colorectal cancer: 4-year follow-up from CheckMate 142 [J]. Ann Oncol 2022;33:1052–60. 35764271 10.1016/j.annonc.2022.06.008

[15] Mantovani A Marchesi F Malesci A. Tumour-associated macrophages as treatment targets in oncology [J]. Nat Rev Clin Oncol 2017;14:399–416. 28117416 10.1038/nrclinonc.2016.217 PMC5480600

[16] DeNardo DG Ruffell B. Macrophages as regulators of tumour immunity and immunotherapy [J]. Nat Rev Immunol 2019;19:369–82. 30718830 10.1038/s41577-019-0127-6 PMC7339861

[17] Daldrup-Link HE Golovko D Ruffell B. MRI of tumor-associated macrophages with clinically applicable iron oxide nanoparticles [J]. Clin Cancer Res 2011;17:5695–704. 21791632 10.1158/1078-0432.CCR-10-3420 PMC3166957

[18] Etzerodt A Tsalkitzi K Maniecki M. Specific targeting of CD163(+) TAMs mobilizes inflammatory monocytes and promotes T cell-mediated tumor regression [J]. J Exp Med 2019;216:2394–411. 31375534 10.1084/jem.20182124 PMC6781002

[19] Toledo B Zhu Chen L Paniagua-Sancho M. Deciphering the performance of macrophages in tumour microenvironment: a call for precision immunotherapy [J]. J Hematol Oncol 2024;17:44. 38863020 10.1186/s13045-024-01559-0 PMC11167803

[20] Zhang L Li Z Skrzypczynska KM. Single-cell analyses inform mechanisms of myeloid-targeted therapies in colon cancer [J]. Cell 2020;181:442–59.e29. 32302573 10.1016/j.cell.2020.03.048

[21] Ginhoux F Schultze JL Murray PJ. New insights into the multidimensional concept of macrophage ontogeny, activation and function [J]. Nat Immunol 2016;17:34–40. 26681460 10.1038/ni.3324

[22] Wenes M Shang M Di Matteo M. Macrophage metabolism controls tumor blood vessel morphogenesis and metastasis [J]. Cell Metab 2016;24:701–15. 27773694 10.1016/j.cmet.2016.09.008

[23] Mulder K Patel AA Kong WT. Cross-tissue single-cell landscape of human monocytes and macrophages in health and disease [J]. Immunity 2021;54:1883–900.e5. 34331874 10.1016/j.immuni.2021.07.007

[24] Zhang Q Liu Y Wang X. Integration of single-cell RNA sequencing and bulk RNA transcriptome sequencing reveals a heterogeneous immune landscape and pivotal cell subpopulations associated with colorectal cancer prognosis [J]. Front Immunol 2023;14:1184167. 37675100 10.3389/fimmu.2023.1184167 PMC10477986

[25] Roelands J van der Ploeg M Ijsselsteijn ME. Transcriptomic and immunophenotypic profiling reveals molecular and immunological hallmarks of colorectal cancer tumourigenesis [J]. Gut 2023;72:1326–39. 36442992 10.1136/gutjnl-2022-327608 PMC10314051

[26] Zhang S Peng W Wang H. C1q(+) tumor-associated macrophages contribute to immunosuppression through fatty acid metabolic reprogramming in malignant pleural effusion. J Immunother Cancer 2023;11:e007441. 10.1136/jitc-2023-007441 PMC10445384 37604643

[27] Sung H Ferlay J Siegel RL. Global cancer statistics 2020: GLOBOCAN estimates of incidence and mortality worldwide for 36 cancers in 185 countries [J]. CA Cancer J Clin 2021;71:209–49. 33538338 10.3322/caac.21660

[28] Bray F Ferlay J Soerjomataram I. Global cancer statistics 2018: GLOBOCAN estimates of incidence and mortality worldwide for 36 cancers in 185 countries [J]. CA Cancer J Clin 2018;68:394–424. 30207593 10.3322/caac.21492

[29] Dekker E Tanis PJ Vleugels JLA. Colorectal cancer [J]. Lancet 2019;394:1467–80. 31631858 10.1016/S0140-6736(19)32319-0

[30] Siegel RL Miller KD Jemal A. Cancer statistics, 2020 [J]. CA Cancer J Clin 2020;70:7–30. 31912902 10.3322/caac.21590

[31] Biller LH Schrag D. Diagnosis and treatment of metastatic colorectal cancer: a review [J]. Jama 2021;325:669–85. 33591350 10.1001/jama.2021.0106

[32] Wei C Yang C Wang S. Crosstalk between cancer cells and tumor associated macrophages is required for mesenchymal circulating tumor cell-mediated colorectal cancer metastasis [J]. Mol Cancer 2019;18:64. 30927925 10.1186/s12943-019-0976-4 PMC6441214

[33] Zuo C Xia J Xu Y. stClinic dissects clinically relevant niches by integrating spatial multi-slice multi-omics data in dynamic graphs [J]. Nat Commun 2025;16:5317. 40523901 10.1038/s41467-025-60575-x PMC12170857

[34] Ma RY Black A Qian BZ. Macrophage diversity in cancer revisited in the era of single-cell omics [J]. Trends Immunol 2022;43:546–63. 35690521 10.1016/j.it.2022.04.008

[35] Li Y Chen Z Han J. Functional and therapeutic significance of tumor-associated macrophages in colorectal cancer [J]. Front Oncol 2022;12:781233. 35186730 10.3389/fonc.2022.781233 PMC8847181

